# LogEvent2vec: LogEvent-to-Vector Based Anomaly Detection for Large-Scale Logs in Internet of Things

**DOI:** 10.3390/s20092451

**Published:** 2020-04-26

**Authors:** Jin Wang, Yangning Tang, Shiming He, Changqing Zhao, Pradip Kumar Sharma, Osama Alfarraj, Amr Tolba

**Affiliations:** 1 School of Computer and Communication Engineering, Hunan Provincial Key Laboratory of Intelligent Processing of Big Data on Transportation, Changsha University of Science and Technology, Changsha 410114, China; jinwang@csust.edu.cn (J.W.); tee@stu.csust.edu.cn (Y.T.); zhaochangqing@stu.csust.edu.cn (C.Z.); 2 Key Lab of Broadband Wireless Communication and Sensor Network Technology (Nanjing University of Posts and Telecommunications), Ministry of Education, Nanjing 210003, China; 3 Department of Computing Science, University of Aberdeen, Aberdeen AB243FX, UK; pradip.academic@gmail.com; 4 Computer Science Department, Community College, King Saud University, Riyadh 11437, Saudi Arabia; oalfarraj@ksu.edu.sa (O.A.); atolba@ksu.edu.sa (A.T.); 5 Mathematics and Computer Science Department, Faculty of Science, Menoufia University, Menoufia 32511, Egypt

**Keywords:** log anomaly detection, word2vec, log event, log template, device management, IoT

## Abstract

Log anomaly detection is an efficient method to manage modern large-scale Internet of Things (IoT) systems. More and more works start to apply natural language processing (NLP) methods, and in particular word2vec, in the log feature extraction. Word2vec can extract the relevance between words and vectorize the words. However, the computing cost of training word2vec is high. Anomalies in logs are dependent on not only an individual log message but also on the log message sequence. Therefore, the vector of words from word2vec can not be used directly, which needs to be transformed into the vector of log events and further transformed into the vector of log sequences. To reduce computational cost and avoid multiple transformations, in this paper, we propose an offline feature extraction model, named LogEvent2vec, which takes the log event as input of word2vec to extract the relevance between log events and vectorize log events directly. LogEvent2vec can work with any coordinate transformation methods and anomaly detection models. After getting the log event vector, we transform log event vector to log sequence vector by bary or tf-idf and three kinds of supervised models (Random Forests, Naive Bayes, and Neural Networks) are trained to detect the anomalies. We have conducted extensive experiments on a real public log dataset from BlueGene/L (BGL). The experimental results demonstrate that LogEvent2vec can significantly reduce computational time by 30 times and improve accuracy, comparing with word2vec. LogEvent2vec with bary and Random Forest can achieve the best F1-score and LogEvent2vec with tf-idf and Naive Bayes needs the least computational time.

## 1. Introduction

Internet of Things (IoT) [1,2] has provided the possibility of easily deploying tiny, cheap, available, and durable devices, which are able to collect various data in real time, with continuous supply [3,4,5,6,7]. IoT devices are vulnerable and usually deployed in harsh and extreme natural environments, thus solutions that can improve monitoring services and the security of IoT devices are needed [8,9,10]. Most smart objects can accumulate log data obtained through sensors during operation. The logs record the states and events of the devices and systems, thus providing a valuable source of information which can be exploited both for research and industrial purposes. The reason is that a large amount of log data stored in such devices can be analyzed to observe user behavior patterns or detect errors in the system. Based on log analysis, better IoT solutions can be developed or updated and presented to the user [11]. Therefore, logs are one of the most valuable data sources for device management, root cause analysis, and IoT solutions updating. Log analysis plays an important role in IoT system management to ensure the reliability of IoT services [12]. Log anomaly detection is a part of log analysis that analyzes the log messages to detect the anomalous state caused by sensor hardware failure, energy exhaustion, or the environment [13].

Logs are semi-structured textual data. An important task is that of anomaly detection in log [14], which is different from the classification and detection in computer vision [15,16,17,18], digital time serial [19,20,21,22,23], and graph data [24]. In fact, the traditional ways of dealing with anomalies in logs are very inefficient. Operators manually check the system log with regular expression matching or keyword searching (for example, “failure”, “kill”) to detect anomaly, which is based on their domain knowledge. However, this kind of anomaly detection is not applicable to large-scale systems.

Many existing works propose schemes to process the logs automatically. Log messages are free-form texts and semi-structured data which should turn into structured data for further analysis. Log parsing [25,26,27] extracts the structured or constant part from log messages. The constant part is named by the *log template* or *log event*. For example, a log message is “*CE sym 2, at 0x0b85eee0, mask 0x05*”. The log event of the log message is *“CE sym <∗>, at <∗>, mask <∗>”*.

Although log events are structured, they are still text data. Most machine learning models for anomaly detection are not able to handle text data directly. Therefore, to extract features of the log event or derive a digital representation of it is a core step. According to the feature extraction results, several machine learning models are used for anomaly detection, such as Regression, Random Forest, Clustering, Principal Component Analysis (PCA), and Independent Component Analysis (ICA) [28]. At first, many statistical features of log event [29,30] are extracted, such as sequence, frequency, surge, seasonality, event ratio, mean inter-arrival time, mean inter-arrival distance, severity spread, and time-interval spread.

More and more works start to apply natural language processing (NLP) methods for the log event vectorization, such as bag-of-words [31], term frequency-inverse document frequency (tf-idf) [32,33] and word2vec [34,35]. Most of the above works are based on the word. Anomalies in logs mostly depend on the log message sequence. Meng et al. [32] form the log event vector by the frequency and weights of words. The log event vector is transformed into the log sequence vector as the input of the anomaly detection model. The transformation from word vector to log event vector or log sequence vector is called *coordinate transformation*. The frequency and weight of words ignore the relevance between words. Bertero et al. [34] detect the anomaly based on the word vector from word2vec [36], which is an efficient method to extract the relevance between words. The word vector is converted to the log event vector, and then the log event vector is converted to the log sequence vector before anomaly detection. However, the computing cost of training word2vec is high and it needs to transform the word vector twice.

As the systems become increasingly complex, there is a large amount of log data. The number of words in each log message is in the range from 10 to 102. Processing words directly is not suitable for large-scale log anomaly detection. Therefore, He et al. [31] propose to count the occurrence number of log events to obtain log sequence vectors directly. The coordinate transformation is unnecessary. In addition, the number of log events is far less than the number of words. The length of the vector is based on the number of words or log events. The dimension of the vector is shortened, which further reduces the computational cost. However, the frequency of log events ignores the relevance of log events.

Therefore, to extract the relevance between log events, reduce the computational cost, and avoid multiple transformations, we investigate the log anomaly detection problem by word2vec with log events as input. The main contributions can be summarized as follows:

- We propose an offline low-cost feature extraction model, named LogEvent2vec, which first takes log events as input of the word2vec model to vectorize the log event vector directly. The relevance between log events can be extracted by word2vec. Only one coordinate transformation is necessary to get the log sequence vector from the log event vector, which decreases the number of coordinate transformations. Training log events is more efficient because the number of log events is less than that of words, which reduces the computational cost.
- LogEvent2vec can work with any coordinate transformation methods and anomaly detection models. After getting the log event vector, the log event vector is transformed into the log sequence vector by bary or tf-idf. Three kinds of supervised models (Random Forests, Naive Bayes, and Neural Networks) are trained to detect the anomaly.
- We have conducted extensive experiments on a real public log dataset from BlueGene/L (BGL). The experimental results demonstrate that our proposed LogEvent2vec can significantly reduce computational time by 30 times and improve the accuracy of anomaly detection, comparing with word2vec.
- Among different coordinate transformation methods and anomaly detection models, LogEvent2vec with bary and Random Forest can achieve the best F1-score and LogEvent2vec with tf-idf and Naive Bayes needs the least computational time. Tf-idf is weaker than bary in aspect of accuracy, but it can significantly reduce the computational time.

The rest of the paper is organized as follows. We introduce the related work in Section 2, and present the general framework of log anomaly detection and the formulation of our work in Section 3. We further provide an overview of our scheme, the log parsing, feature extraction, and anomaly detection model in Section 4. Finally, we evaluate the performance of the proposed algorithms through extensive experiments in Section 5 and conclude the work in Section 6.

## 2. Related Work

According to the framework of log anomaly detection in Section 3, log anomaly detection consists of several important steps. We review the related works for each step.

### 2.1. Log Parsing

Log parsing extracts the log template or log event from the raw log. A log template is a log event that records events occurring in the execution of a system. FT-tree [25] identifies the longest combination of frequently occurring words as a log template. He et al. [26] design and implement a parallel log parser (namely POP) on top of Spark, a large-scale data processing platform. The raw log is divided into constant and variable, and the same log events are combined into the same clustering group by hierarchical clustering. He et al. also propose an online log parsing method, namely Drain [27], which uses a fixed depth parse tree to accelerate parsing. He et al. [37] provide the tools and benchmarks for automated log parsing.

### 2.2. Feature Extraction

Extracting the feature of logs is the basis of anomaly detection. Zhang et al. [29] propose Prefix to extract four features (sequence, frequency, surge, seasonality) from the log sequence and form a feature matrix. Khatuya et al. [30] select features from system logs, including event count, event ratio, mean inter-arrival time, mean inter-arrival distance, severity spread, and time-interval spread, and transform the log events into score matrix. Liu et al. [38] extract 10 features and compress to two features.

In addition, the NLP methods start to attract the researcher’s interest to vectorize the log event, such as bag-of-words [39], TF-IDF [40], and word2vec.

He et al. [31] count the occurrence number of each log event to form the event count vector for each log sequence, whose basic idea draws from bag-of-words. Meng et al. [32] propose LogClass which combines a word representation method, named tf-idf, with the Positive-unlabeled (PU) learning model to construct device-agnostic vocabulary with partial labels. Lin et al. [33] propose an approach named LogCluster which turns each log sequence into a vector by Inverse Document Frequency (IDF) and Contrast-based Event Weighting.

Bertero et al. [34] consider logs as regular text and first apply a word embedding technique based on Google’s word2vec algorithm, in which logfiles’ words are mapped to a high dimensional metric space. Then, the coordinate of the word is transformed into the log event vector, and the coordinate of the log event vector is transformed into the log sequence vector. Meng et al. [35] propose LogAnomaly, a framework to model a log stream as a natural language sequence. They propose a novel, simple feature extraction method, template2vec, to extract the semantic information hidden in log templates by a distributional lexical-contrast embedding model (dLCE) [41]. The word vector is transformed to the log event vector, which is fed into the long short-term memory (LSTM) detection model.

According to the type of anomaly detection, the word vector from word2vec needs to form the log event vector or the log sequence vector. For example, the log event vector is enough for LSTM [35], while the log sequence vector is needed for Random Forest or Naive Bayes [34].

Table 1 concludes the NLP methods on log feature extraction. To avoid multiple transformations, the objects of NLP methods become log events from words. Therefore, this paper handles the log events directly.

### 2.3. Anomaly Detection

After feature extraction, several machine learning models are used for anomaly detection, such as Regression [30], Random Forest [29,32], and Clustering [33,38,42].

Ridge regression is used to estimate the abnormal score from the features [30], and the total weight vector obtained by ridge regression is used for express the relative importance of different features. Random Forest is used to anomaly detection based on the feature matrix in Prefix [29]. LogClass [32] classifies anomalies based on device logs by Random Forest.

LogCluster [33] clusters the logs to ease log-based problem identification, which utilizes a knowledge base to check if the log sequences occurred before. Liu et al. [38] make use of a mixed attribute clustering method k-prototype, which transforms data from 10 features to a new data set to reduce feature dimensions. Then, k-Nearest Neighbor (k-NN) classifier is used to identify the real abnormalities in the new data set, which greatly reduces the calculation scale and time. Loglens [42] is a real-time log analysis system, which clusters log events by similarity measure.

A comparison among six state-of-the-art log-based anomaly detection methods is presented in [31], including three supervised methods (Logistic Regression, Decision Tree, and Support Vector Machine (SVM)) and three unsupervised methods (LogCluster, PCA, Invariant Mining), and an open-source toolkit allowing ease of reuse.

In addition, deep learning methods [43] are applied in log anomaly detection [35]. Deeplog [44] uses LSTM to model a certain type of log key sequence of logs, automatically learns the normal mode from the normal log data, and then judges system exceptions. Refs [45,46] analyze the application of various LSTM models in anomaly detection, such as bidirectional LSTM, stacked LSTM, etc.

In this paper, we show that our feature extraction algorithm can work well with various anomaly detection methods.

## 3. General Framework and System Model

**Figure 1:**
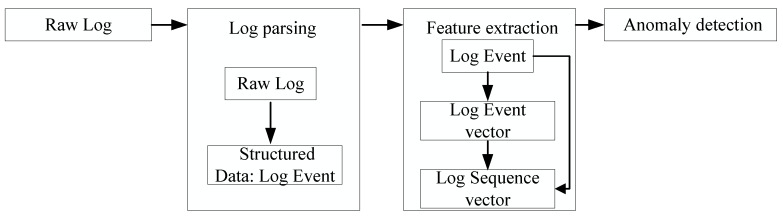
The framework of log anomaly detection.

In this section, we introduce the general framework of log anomaly detection and the formulation of our work. The general framework of log anomaly detection consists of three steps: log parsing, feature extraction, and anomaly detection, as shown in Figure 1. Table 2 summarizes the notations and definitions used in this paper.

### 3.1. Log Parsing

Logs are semi-structured. A log message can be divided into two parts: a constant part and a variable part (some specific parameters). A log event is the template (constant part) of a log message. To turn semi-structured raw logs into structured data, log parsing extracts a set of templates to record events that occur during the execution of a system. In this paper, we do not distinguish between the log template and the log event.

The log data from a system are denoted by *L*. The log data contain *N* lines of log messages. The *i*th log message is denoted by $l_{i}\in L,1\leq i\leq N$. Every log message is generated by an application of the system to report an event. Every log message consists of a list of words, similar to a sentence.

The log parsing [27] is used to remove all specific parameters from log messages and extract all the log events. The set of log events is denoted by *E*, in which the number of log events is *M*. In this way, each log message is mapped into a log event. Log parsing can be represented by the mapping function *p*. The log event of the log message $l_{i}$ can be described as $p(l_{i})\in E$:

(1) p:L→E

Then, log data are divided into various chunks. A chunk is a log sequence. We assume that the fixed window is used and the window size decides the length of log sequences, denoted by *W*. There are N/W log sequences, where the set of log sequences is denoted by LSE. The *i*th sequence consists of *W* log messages from $l_{iW+1},l_{iW+2}$, to $l_{iW+W}$. Each log message in a log sequence can be mapped into a log event [47]. As a result, the log sequence can be treated as a list of log events. The log sequence $lse_{i}$ is denoted by

(2) $$\begin{array}{c} lse_{i}=\left[p\left(l_{iW+1}\right),p\left(l_{iW+2}\right),\ldots ,p\left(l_{iW+W}\right)\right],0\leq i\leq N/W-1,lse_{i}\in LSE. \end{array}$$

### 3.2. Feature Extraction

Although log events are structured, they still consist of text. Therefore, the log event should be numerically encoded for further anomaly detection. Text of log events can be encoded by NLP models. The list of logs are divided into various chunks, which are log sequences. A feature vector is generated to represent a log sequence.

Word2vec [36] is used to extract features of log events. Generally speaking, word2vec maps words of a text corpus into a Euclidean space. In the Euclidean space, relevant words are close, while irrelevant words are far away.

In our case, we use word2vec to map log events of log sequence into a Euclidean space. The input of word2vec is a list of log events instead of a list of words. Thus, every log event gets a coordinate, denoted by v(e),e∈E in a vector space *T*. After mapping each log event, a log sequence can be represented by a function of its all log events’ coordinates. It means that each log sequence is also mapped into the vector space. The mapping of log event and log sequence can be represented as two functions:

(3) $$\begin{array}{c} v:E\to T\\ f:LSE\to T \end{array}.$$

According to the definition of log sequence in Equation (2), the log events of log sequence $lse_{i}$ are $p\left(l_{iW+1}\right),p\left(l_{iW+2}\right),\ldots ,p\left(l_{iW+W}\right)$. The coordinate of the log event related to the log message $l_{j}$ can be denoted by $v(p\left(l_{j}\right))$. Therefore, the coordinates of these log events are $v\left(p\left(l_{iW+1}\right)\right),v\left(p\left(l_{iW+2}\right)\right),\ldots ,v\left(p\left(l_{iW+W}\right)\right)$. By the above-described procedure, the coordinate of the log sequence depends on all its log events’ coordinates. The log sequence $lse_{i}$ can be assigned to a coordinate by $f\left(lse_{i}\right)=f\left(\left[p\left(l_{iW+1}\right),p\left(l_{iW+2}\right),\ldots ,p\left(l_{iW+W}\right)\right]\right)=f\left(v\left(p\left(l_{iW+1}\right)\right),v\left(p\left(l_{iW+2}\right)\right),\ldots ,v\left(p\left(l_{iW+W}\right)\right)\right)$.

### 3.3. Anomaly Detection

All feature vectors of log sequence are the samples, which are trained for machine learning or deep learning models to detect anomaly. Then, the trained model predicts whether a new log sequence is anomalous or not.

A binary classifier *c* is trained on $f(lse_{i}|lse_{i}\in LSE)\in T$. This kind of classifier *c* can be treated as an ideal separation function: c:T→[0,1]. The classifier determines whether a log sequence $lse_{i}$ is anomalous (label $y_{i}=1$ denotes an anomalous log sequence and $y_{i}=0$ denotes a normal log sequence) or not. When the anomalous event occurs, the log message at that time is labeled anomalous. If an anomalous log message belongs to a log sequence, this log sequence is labeled as an anomaly. Otherwise, the log sequence is normal when all log messages in it are normal. In the case log sequence contains log events which do not occur, those are simply ignored.

## 4. Methodology

**Figure 2:**
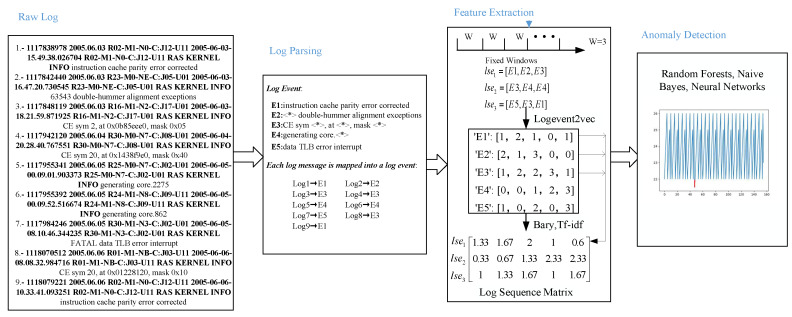
Overview of Log Event to vector based log anomaly detection.

The overview of LogEvent-to-vector based log anomaly detection is shown in Figure 2. The first block shows nine raw logs in the BGL dataset. The second block is the log parsing step which extracts five log events from the raw logs by the Drain. Each log is mapped into a log event. The third block is the feature extraction step. Logs are divided into log sequences by a fixed window. Each log event vector is obtained by logEvent2vec which takes the log event as the processing object. The log sequence vector is calculated by all log event vectors in the log sequence according to bary or tf-idf. The fourth block is the anomaly detection. The anomalies are marked by the red line. Three kinds of supervised models (Random Forests, Naive Bayes, and Neural Networks) are trained to detect the anomaly. The detailed process of each step is described below.

### 4.1. Log Parsing

There are nine raw log messages of BGL in the first block of Figure 2. Each log message contains timestamp, date, node, time, node repeat, message type, component (message generation location), level, and content. For example, the third log message is “*1117848119 2005.06.03 R16-M1-N2-C:J17-U01 2005-06-03-18.21.59.871925 R16-M1-N2-C:J17-U01 RAS KERNEL INFO CE sym 2, at 0x0b85eee0, mask 0x05*”. *1117848119* is the time stamp, *2005.06.03* is the data, *R16-M1-N2-C:J17-U01* is the node, *2005-06-03-18.21.59.871925* is the time, *R16-M1-N2-C:J17-U01* is the node repeat, *RAS* is the message type, *KERNEL* is the component, *INFO* is the level, and *CE sym 2, at 0x0b85eee0, mask 0x05* is the content.

After parsing by Drain [27], the log event is shown in the last row of Table 3. The semi-structured raw log message is converted into structured information. The variable part in the log message is replaced by a wildcard, and the constant part remains unchanged. Each log event has a unique log event and event template. The event template of the third log message is *“CE sym <∗>, at <∗>, mask <∗>”* with log event E3 as shown in the second block of Figure 2. Similarly, we get five log events E1–E5 in the second block from the nine raw log messages. Each raw log message is mapped into a log event. For example, the first log message is mapped into log event E1, and the second log message is mapped into log event E2.

### 4.2. Feature Extraction

LogEvent2vec takes the log event as input of the word2vec model, and then transforms the log event vector to the log sequence vector. Because the number of log events is far less than the number of words, LogEvent2vec reduces the training cost. In addition, only one coordinate transformation is necessary to get the log sequence vector from the log event vector.

#### 4.2.1. LogEvent2vec: Log Event Training Via Word2vec

Word2vec maps words to vectors, which is divided into two models, namely continuous skip-gram model (skip-gram) and continuous bag-of-words model (cbow) [48]. The training input of cbow model is the context word vector of a target word, and the output is the word vector of the target word. The idea between skip-gram and cbow is opposite, that is, the input is the word vector of a target word, and the output is the context word vector of the target word. Cbow model is used in this paper.

**Figure 3:**
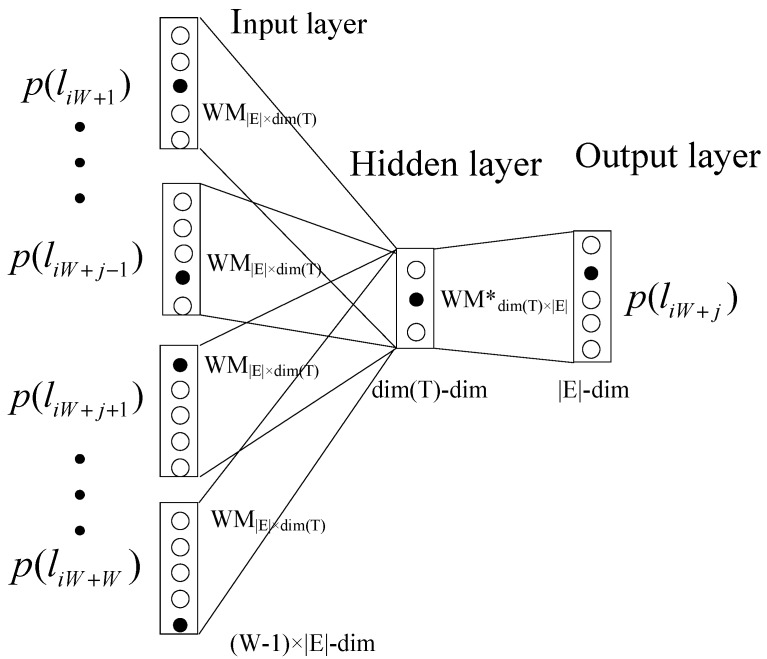
Log event as input of the word2vec model. The target is log event $p(l_{iW+j})$, and the rest log events $p\left(l_{iW+1}\right),p\left(l_{iW+2}\right),\ldots ,p\left(l_{iW+j-1}\right),p\left(l_{iW+j+1}\right),\ldots ,p\left(l_{iW+W}\right)$ in the log sequence $lse_{i}$ are taken as input.

Cbow model consists of three layers [49]: input layer, hidden layer, and output layer, as shown in Figure 3. For example, the corpus is “I drink coffee every day”. We can get the embedding of “coffee” from the rest four words “I”, “drink”, “every”, and “day” which are taken as input. Similarly, we can get the embedding of all words.

LogEvent2vec takes the log event as the input of word2vec to get the embedding of each log event in vector space *T*. The space dimension is dim(T). If the target is log event $p(l_{iW+j})$, the rest log events $p\left(l_{iW+1}\right),p\left(l_{iW+2}\right),\ldots ,p\left(l_{iW+j-1}\right),p\left(l_{iW+j+1}\right),\ldots ,p\left(l_{iW+W}\right)$ in the log sequence $lse_{i}$ are taken as input, as shown in Figure 3. For example, we assume that the fixed window size is 3, as shown in the third block of Figure 2. The nine log messages are divided into three sequences ($lse_{1},lse_{2},lse_{3}$) which are [E1,E2,E3],[E3,E4,E4], and [E5,E3,E1], respectively. LogEvent2vec takes log events E1 and E3 in the first sequence as the input and log event E2 as the output. Similarly, log events E3 and E4 in the second sequence are taken as the input of word2vec while the target is log event E4. Log events E5 and E1 in the third sequence are taken as the input of word2vec while the target is log event E3.

In detail, the one-hot vector with |E| dimension is used to represent the log event. There are W−1 one-hot vectors in the input layer. The output layer is the one-hot vector of the target log event. The hidden layer’s dimension is dim(T). After training the model, we can get the embedding of a log event by multiplying its one-hot vector and the weight matrix $WM\in R^{|E|\times dimT}$. Assuming that the dimension is set to 5, the embedding vectors of log events E1−E5 are [1,2,1,0,1], [2,1,3,0,0], [1,2,2,3,1], [0,0,1,2,3], [1,0,2,0,3], as shown in the third block of Figure 2.

#### 4.2.2. From Log Event Vector to Log Sequence Vector

All log event vectors in space *T* are produced by LogEvent2vec. To get the log sequence vector in space *T*, we transform log event vector to log sequence vector by bary or tf-idf:

- Bary defines the vector of log sequence as the average of all its log events in Equation (4):
  (4) $$\begin{array}{c} f\left(lse_{i}\right){=}_{def}1/\left|lse_{i}\right|\sum_{k=1}^{k=W}v\left(p\left(l_{iW+k}\right)\right),lse_{i}\in LSE. \end{array}$$
- Tf-idf defines the vector of log sequence as the weighted average of all its log events. The weight depends on the frequency of log events. A rare log event has a higher weight than a frequent log event.

According to bary, the vector of first log sequence $lse_{1}$ is the average position of E1,E2,E3, which is ([1,2,1,0,1]+[2,1,3,0,0]+[1,2,2,3,1])/3=[1.33,1.67,2,1,0.6], as shown in the third block of Figure 2. Similarly, we can calculate the vectors of $lse_{2}$ and $lse_{3}$, which are [0.33,0.67,1.33,2.33,2.33], [1,1.33,1.67,1,1.67], respectively.

After transformation, we can obtain all log sequences’ vector, which is a matrix with N/W×dim(T).

### 4.3. Anomaly Detection

Anomaly detection can be treated as a binary classification problem. Many classifiers are available. In this paper, we use three supervised algorithms to detect anomaly: Random Forests, Naive Bayes, and Neural Networks in this part. The log sequence matrix is the input of the anomaly detection model.

## 5. Evaluation

### 5.1. Datasets

To evaluate the performance of our proposed algorithms, we use the BGL dataset from the BlueGene/L supercomputer system at Lawrence Livermore National Labs (LLNL) [50]. Table 4 shows the basic information of the BGL dataset. There are 4,747,963 log messages and 348,460 anomalous log messages in the BGL dataset.

### 5.2. Experimental Setup

All experiments are run on Baidu AI Studio (Beijing, China), which provides a server with an Intel(R) Xeon(R) Gold 6148 CPU (Beijing, China) with 8 core, NVIDIA Tesla V100 with 16 GB VideoMem GPU (Beijing, China), and 32 GB RAM.

After Drain [27] log parsing, we obtain 376 log events. By default, according to [34], the window size of fixed windows is set to 5000 and the dimension of vector dim(T) is set to 20. It means that the length of each log sequence is 5000. After dividing, there are 943 log sequences. We randomly choose the 90% log sequence as the training data, and the remaining 10% as the testing data. All results are averages of five times results.

We compare our feature extraction scheme with the method in [34] with different coordinate transformation and anomaly detection models. The two kinds of feature extraction schemes have two kinds of coordinate transformations: bary and tf-idf. There are three kinds of supervised methods: Random Forests, Naive Bayes, and Neural Networks. The two kinds of feature extraction schemes are described as follows:

- Word [34]: It takes words as input of the word2vec model after removing the non-alphanumeric characters. After getting the words vector, it performs coordinate transformation twice to get the log file vector.
- LogEvent: our approach takes log events as input of the word2vec model after log parsing. After getting the log event vector, it performs coordinate transformation once to get the log sequence vector.

As shown in Table 5, we have 12 kinds of schemes. We use three combined characters to represent the schemes. For example, “W-b-NB” means the method in [34] with two bary coordinate transformations and Naive Bayes anomaly detection model. “LE-t-NN” means our approach with a tf-idf coordinate transformation and Neural Networks anomaly detection model. The implementations of tf-idf, Random Forests, Naive Bayes, and Neural Networks are from the scikit-learn (http://scikit-learn.org/) standard library.

F1-score, Area Under Curve (AUC), and computational time are used to evaluate the accuracy of anomaly detection methods. F1-score is an index used to measure accuracy of binary classification model in statistics. It takes into account both accuracy and recall of classification model. F1-score can be regarded as the harmonic average of precision and recall in Equation (5). It has its best value at 1 and worst at 0. AUC is a kind of evaluation index to measure the quality of the binary classification model, which indicates the probability that the positive example prediction is in front of the negative example. Computational time includes the time of feature extraction, the time of training anomaly detection model, the time of issuing all predictions in the test set, and the total time. The computational time of feature extraction consists of training word2vec and coordinate transformations. The total time is from word2vec training to the anomaly detection model without preprocessing because of the different preprocessing in our scheme and [34]:

(5) $$\begin{array}{c} F=2\times precision\times recall/(precision+recall) \end{array}$$

### 5.3. Experiment Results

#### 5.3.1. Impact of Anomaly Detection Model

To investigate the effect of different anomaly detection models, we analyze the F1-score, AUC, and computational time of Random Forests, Naive Bayes, and Neural Networks, while other parameters are set to the default values and the coordinate transformation method is bary.

**Figure 4:**
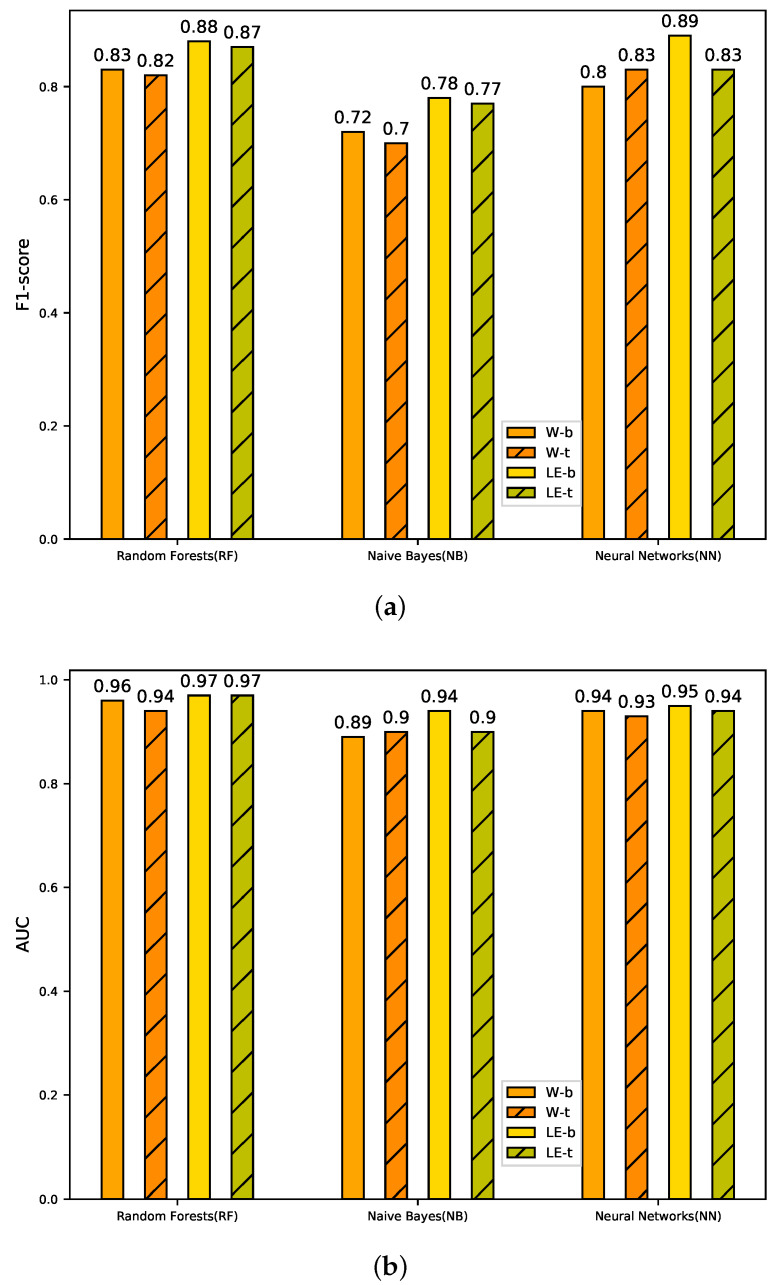
Performance of different schemes with bary and tf-idf coordinate transformations. (**a**) F1-score; (**b**) AUC.

The results are as shown in Figure 4. In the aspect of the detection model, we can see that the anomaly detection performance of W-b-RF (F1-score = 0.83, AUC = 0.96) and W-b-NN (F1-score = 0.80, AUC = 0.94) are far higher than that of W-b-NB (F1-score = 0.72, AUC = 0.89). In the case of log event as input, the detection performance of LE-b-RF (F1-score = 0.88, AUC = 0.97) and LE-b-NN(F1-score = 0.89, AUC = 0.95) is better than that of LE-b-BN (F1-score = 0.78, AUC = 0.94). The detection performance of Random Forest and Neural Network as classifier is better than Naive Bayes. The reason is that Random Forest is a set of decision trees, in which each decision tree processes samples and predicts output labels. The decision trees in the set are independent, and each decision tree can predict the final result. Neural Networks are fully connected. They are grouped by layers and process the data in each layer and deliver it to the next layer. The last layer of neurons is responsible for the prediction. Therefore, those two detection models consider the relevance between features. The premise of Naive Bayes algorithm is that the features are independent. There is a certain relevance between the log events in the log sequence. Therefore, Random Forest and Neural Network are better than Naive Bayes.

In the aspect of the input, the results of anomaly detection using log events as input are better than using words as input. The AUC score of LE-b-RF is 0.97. The reason is that the representation of the log sequence vector is more accurate in LogEvent2vec. Inputting words to word2vec [34] needs to transform word vectors into the vector of log sequence by two coordinate transformations, so there will be some bias in the representation of the log sequence vector and affect the final anomaly detection results. Our schemes only need to perform one coordinate transformation to get the log sequence vector. Therefore, the log sequence vector is more accurate in representation. Logevent2vec reduces the number of coordinate transformations and obviously improves its F1-score. The results confirm the rationality of LogEvent2vec.

**Figure 5:**
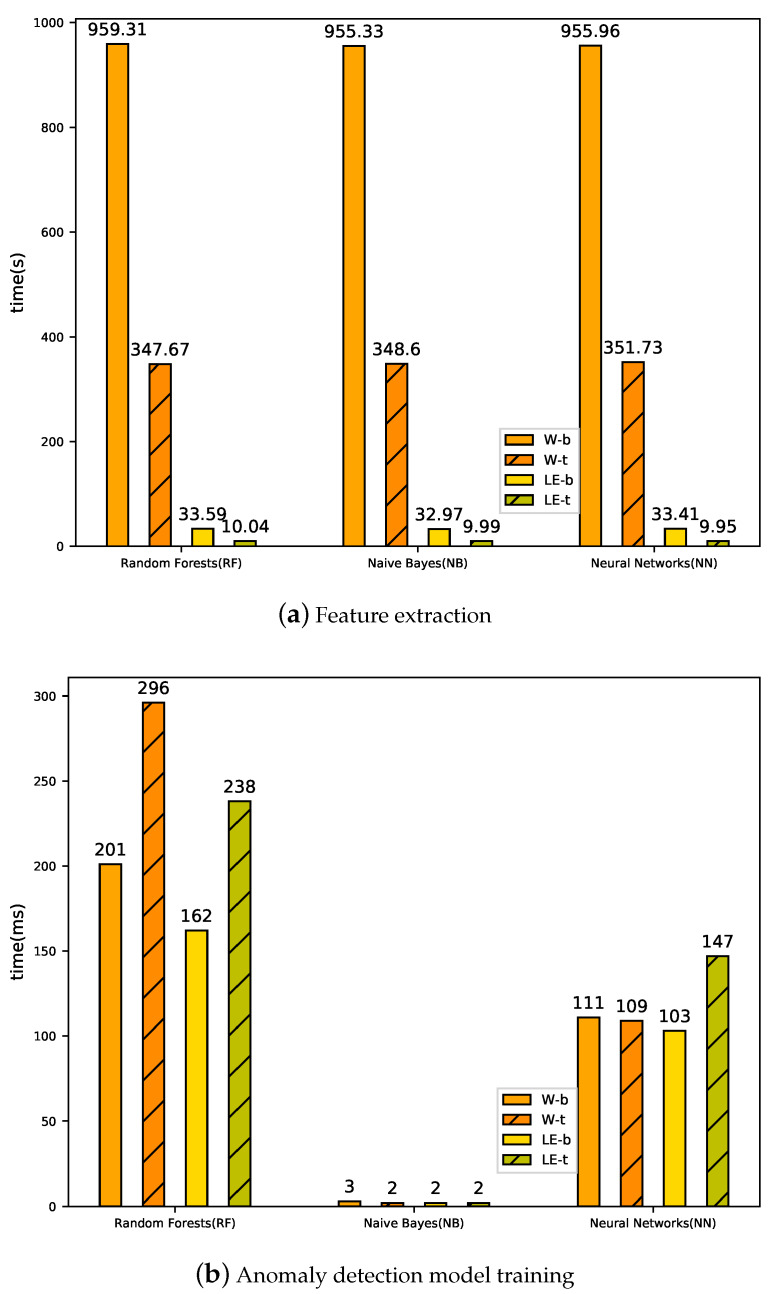
Computational time of the feature extraction and anomaly detection with bary and tf-idf coordinate transformations. (**a**) computational time of feature extraction; (**b**) computational time of anomaly detection model training.

**Figure 6:**
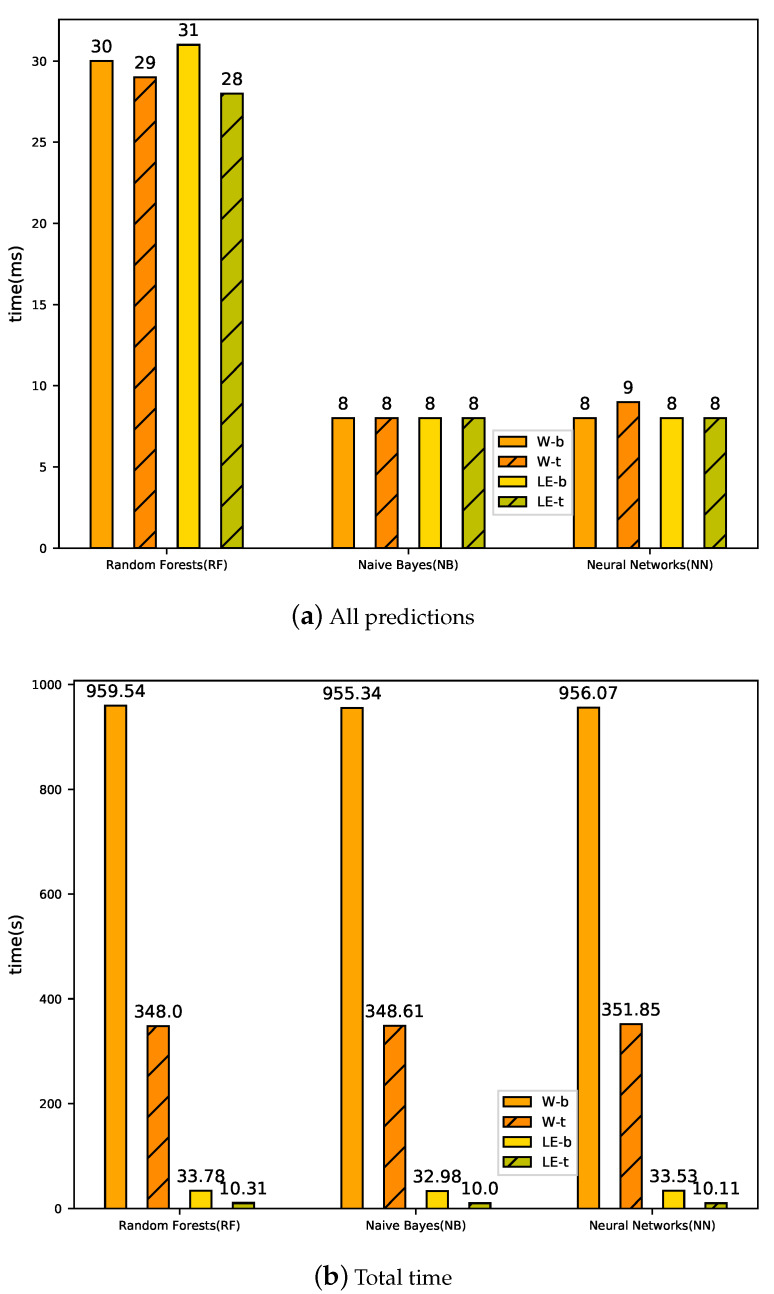
Computational time of training and testing with bary and tf-idf coordinate transformations. (**a**) computational time of issuing all predictions in test set; (**b**) total computational time.

Figure 5 and Figure 6 show the computation time for three classifiers to detect anomaly. Figure 5a shows the time of feature extraction from training word2vec to coordinate transformation, where training word2vec consumes the majority of the time. It can be seen that the time required for feature extraction in Random Forest is the highest, and the time required for feature extraction in Naive Bayes is the lowest. The number of words in the BGL dataset is 1,405,168, while the number of log events is only 376. The training time of log events in word2vec is far less than that of words. The final experimental results show that LogEvent2vec (32.97 s < 955.33 s) takes less time to train word2vec.

Figure 5b and Figure 6a show the time needed to train the classifier with 848 log sequences and issue 95 log sequences for prediction. We can see that LogEvent2vec and word2vec consume the same time in training the classifier and issuing the test set for prediction. Figure 6b shows the total time from training word2vec to finally completing anomaly detection. The total time is mainly determined by the time of feature extraction: the less time word2vec training takes, the less time it takes. Finally, the experiment shows that LogEvent2vec shortens time by 30 times than word2vec.

#### 5.3.2. Impact of Coordinate Transformation

We analyze the performance of bary and tf-idf with other parameters set to the default values. The results are shown in Figure 4.

Taking Random Forest as an example, the performances of W-b (F1-score = 0.83, AUC = 0.96), W-t (F1-score = 0.82, AUC = 0.94), LE-b (F1-score = 0.88, AUC = 0.97), and LE-t (F1-score = 0.87, AUC = 0.97) are not very different from each other. The results of other anomaly detection models are similar. The way of coordinate transformation (bary and tf-idf) has little influence on the result of the anomaly detection.

However, different inputs of word2vec have a great influence on anomaly detection results. We can see that the method of LogEvent2vec (LE-b (F1-score = 0.88, AUC = 0.97), LE-t (F1-score = 0.869, AUC = 0.97)) is better than word2vec (W-b (F1-score = 0.834, AUC = 0.959), W-t (F1-score = 0.825, AUC = 0.944)) with the Random Forest model.

Figure 5 and Figure 6 show the time consumed with tf-idf coordinate transformations. The computational time of feature extraction in W-t-NN is the longest (351.73 s) and LE-t-NN is the shortest (9.95 s), as shown in Figure 5a. In addition, the computational time of feature extraction in W-b-RF is 959.31 s, while that in W-t-RF is only 347.67 s. It can be seen that tf-idf can reduce the time consumption in feature extraction. Figure 5b and Figure 6a show the time consumed by training the anomaly detection model and issuing all predictions in the test set. It can be seen that different coordinate transformations have less impact on the time consumed by training the anomaly detection model and issuing predictions. Figure 6b shows the total consumption time of anomaly detection with tf-idf coordinate transformation, which is still mainly determined by the computational time of feature extraction. Therefore, the performance of tf-idf is weaker than that of bary, but tf-idf can significantly reduce the computational time.

#### 5.3.3. Impact of the Dimension

To investigate the effect of the dimension of feature space, the number of dimensions is set from 5 to 500 while other parameters are set to the default values.

In Table 6 and Table 7, LE-b-NN has the best performance in all classification when dimensions are from 5 to 50. The performance of LE-b-RF is the best when dimensions are from 100 to 500.

Although the AUC score of W-b-RF (F1-score = 0.81) is the highest at 200 dimensions, its F1-score of correct classification is lower than that of LE-b-RF (F1-score = 0.84). The difference of AUC score between LE-b-RF and W-b-RF is 0.008, while the difference of F1-score is 0.03. Therefore, the performance of LE-b-RF is better than that of W-b-RF in 200 dimensions. Generally speaking, we use log events as input of the word2vec model, and the effect of anomaly detection is better than using words as input of the word2vec model. The final experimental results also confirm that the effect of LogEvent2vec is better than that of word2vc.

Table 8, Table 9, Table 10 and Table 11 depict the time of feature extraction, the time of training anomaly detection model, the time of issuing all predictions in the test set, and the total time with bary coordinate transformation, respectively. No matter which kind of anomaly detection models and feature extraction, the time of feature extraction and training the anomaly detection model increases as the dimension increases. Therefore, the total time is also increasing with the increase of dimensions. However, the dimension has less impact on the time of issuing all predictions in the test set.

## 6. Conclusions

We propose LogEvent2vec, an offline feature extraction approach that takes the log event as the input of word2vec to extract the relevance between log events, and reduce the time of training and coordinate transformation. LogEvent2vec can work with any coordinate transformation methods and anomaly detection models. The experimental results demonstrate that our approach is effective and outperforms the state-of-the-art work. Compared with Neural Network and Naive Bayes model, the performance of Random Forest as classifier working with LogEvent2vec is better. Different coordinate transformation methods (bary and tf-idf) have less influence on the accuracy of anomaly detection, but tf-idf can significantly reduce the computational time. LogEvent2vec working with LSTM is our future work.

## Figures and Tables

**Table 1:** Feature Extraction based on NLP.

Method	Word	Log Event
Bag-of-words	Forming the log event vector by the occurrence number of words	Forming the log sequence vector by the occurrence number of the log event [31]
Idf/Tf-idf	Forming the log event vector by the term frequency and weights of words [32]	Forming the log sequence vector by the term frequency and weights of the log event [33]
Word2vec	Forming the word vector by Word2vec [34,35]	–

**Table 2:** List of notations.

Notation	Definition
*L*	The log data
*N*	The number of lines in log data
*E*	The set of log events
*M*	The number of log events
LSE	The set of log sequences
*W*	The window size which decides the length of a log sequence
*T*	The vector space
$l_{i}$	The *i*th log message
p(.)	The mapping function of log parsing
$p(l_{i})$	The log event of log message $l_{i}$
$lse_{i}$	The *i*th log sequence that is $\left[p\left(l_{iW+1}\right),p\left(l_{iW+2}\right),\ldots ,p\left(l_{iW+W}\right)\right]$
v(e)	The vector of log event *e*
$f(lse_{i})$	The prediction of log sequence $lse_{i}$ that is $f(v\left(p\left(l_{iW+1}\right)\right),v\left(p\left(l_{iW+2}\right)\right),\ldots ,v\left(p\left(l_{iW+W}\right)\right))$
$y_{i}$	The label of log sequence $lse_{i}$

**Table 3:** Raw log and log event.

Item	Content
Time stamp	1117848119
Data	2005.06.03
Node	*R16-M1-N2-C:J17-U01*
Time	2005-06-03-18.21.59.871925
Node repeat	*R16-M1-N2-C:J17-U01*
Message type	*RAS*
Component	*KERNEL*
Level	*INFO*
Content	*CE sym 2, at 0x0b85eee0, mask 0x05*
Log event	*“CE sym <∗>, at <∗>, mask <∗>”*

**Table 4:** Summary of BGL dataset.

System	#Time Span	#Data Size	#Log Messages	#AnomaliesLog
BGL	7 months	708M	4,747,963	348,460

**Table 5:** The component of comparison schemes.

Steps	Models
Word2vec input unit	Word/Log event
Coordinate transformation	Bary/Tf-idf
Anomaly detection model	Random Forests/Naive Bayes/Neural Networks

**Table 6:** F1-score with different dimensions.

dim(T)	W-b-RF	W-b-BN	W-b-NN	LE-b-RF	LE-b-BN	LE-b-NN
5	0.821788573	0.638914042	0.599454543	0.848245935	0.654693534	$\underline{0.86068197}$
10	0.826054636	0.715535678	0.707411424	0.827861155	0.745818201	$\underline{0.849900514}$
20	0.834107143	0.72259094	0.803393267	0.879608688	0.782222222	$\underline{0.886912543}$
50	0.785066632	0.732549521	0.80844075	0.877704266	0.776404488	$\underline{0.880865736}$
100	0.814447561	0.751401056	0.747072721	$\underline{0.885832862}$	0.777671294	0.829474969
200	0.811296155	0.70370138	0.808328189	$\underline{0.840172605}$	0.800541113	0.826401595
500	0.761251469	0.72595185	0.766749974	$\underline{0.855822584}$	0.8009675	0.846823786

**Table 7:** AUC with different dimensions.

dim(T)	W-b-RF	W-b-BN	W-b-NN	LE-b-RF	LE-b-BN	LE-b-NN
5	0.952121313	0.893721708	0.851681379	0.966854572	0.888070647	$\underline{0.973852043}$
10	0.946359642	0.910339212	0.883053652	$\underline{0.960652723}$	0.913831019	0.940199701
20	0.959049752	0.88620832	0.940347094	$\underline{0.969534015}$	0.939435145	0.950155508
50	0.936857925	0.909702317	0.932487478	$\underline{0.979603173}$	0.921604084	0.941439031
100	0.952108191	0.918905966	0.912478988	$\underline{0.959295558}$	0.928927259	0.911241156
200	$\underline{0.965241513}$	0.890634611	0.903616499	0.957165279	0.929082466	0.921258002
500	0.939609334	0.869648164	0.882033281	$\underline{0.963988762}$	0.917090636	0.92435774

**Table 8:** Computational time of the feature extraction with different dimensions.

dim(T)	W-b-RF	W-b-BN	W-b-NN	LE-b-RF	LE-b-BN	LE-b-NN
5	928.467184365	928.154774189	928.213744521	33.440349197	$\underline{32.785332489}$	33.096653652
10	950.101264000	949.874471283	948.049470901	33.298034906	$\underline{32.398853445}$	33.481590986
20	959.311112213	955.330675745	955.955496573	33.588190031	$\underline{32.973171473}$	33.414360476
50	963.718184471	965.015199471	964.497252941	34.691981173	33.850553179	$\underline{33.587378836}$
100	1006.996418762	1008.646436644	1009.821412706	33.849840307	$\underline{33.255211973}$	33.166025877
200	1060.622915459	1063.216091013	1070.170886092	35.303260088	$\underline{34.084345007}$	36.286307726
500	1244.848617029	1245.274248505	1252.219076300	40.350662804	$\underline{38.923700237}$	39.244319487

**Table 9:** Computational time of the anomaly detection with different dimensions.

dim(T)	W-b-RF	W-b-BN	W-b-NN	LE-b-RF	LE-b-BN	LE-b-NN
5	0.129946113	0.002494335	0.091389656	0.118011236	$\underline{0.002314472}$	0.092924643
10	0.174678040	0.002696085	0.088878870	0.140994787	$\underline{0.002168465}$	0.098157644
20	0.200891352	0.002576542	0.111003637	0.162158251	$\underline{0.002343321}$	0.103493547
50	0.302109051	0.003390408	0.131638622	0.226914167	$\underline{0.002695179}$	0.140838718
100	0.419996023	0.003537750	0.628135109	0.280465174	$\underline{0.002766132}$	0.576955175
200	0.592480183	0.003967857	1.132779264	0.374702978	$\underline{0.003175211}$	0.917661619
500	0.874522972	0.005608940	1.402945185	0.557642794	$\underline{0.005089426}$	1.660369825

**Table 10:** Computational time of issuing all prediction in test set with different dimensions.

dim(T)	W-b-RF	W-b-BN	W-b-NN	LE-b-RF	LE-b-BN	LE-b-NN
5	0.030201614	$\underline{0.007484496}$	0.007597148	0.030419779	0.008524370	0.008317709
10	0.030166960	0.007774067	0.008397007	0.028655720	$\underline{0.007569742}$	0.008075190
20	0.030082989	0.007681179	0.007875299	0.030849648	$\underline{0.007559681}$	0.008102942
50	0.031879997	0.007841539	0.008520412	0.031287003	$\underline{0.007819939}$	0.007965803
100	0.031735134	0.008501720	0.008821011	0.029254770	$\underline{0.007116508}$	0.007657909
200	0.031595659	0.009186029	0.091766162	0.029769993	0.007659483	$\underline{0.006997528}$
500	0.030633450	0.010237265	0.014094353	0.030339813	0.009008598	$\underline{0.008466959}$

**Table 11:** Total computational time with different dimensions.

dim(T)	W-b-RF	W-b-BN	W-b-NN	LE-b-RF	LE-b-BN	LE-b-NN
5	928.627332091	928.164753020	928.312731326	33.588780212	$\underline{32.796171331}$	33.197896004
10	950.306108999	949.884941435	948.146746778	33.467685413	$\underline{32.408591652}$	33.587823820
20	959.542086554	955.340933466	956.074375510	33.781197929	$\underline{32.983074474}$	33.525956964
50	964.052173519	965.026431417	964.637411976	34.950182343	33.861068296	$\underline{33.736183357}$
100	1007.448149920	1008.658476114	1010.458368826	34.159560251	$\underline{33.265094614}$	33.750638962
200	1061.246991301	1063.229244900	1071.395431519	35.707733059	$\underline{34.095179701}$	37.210966873
500	1245.753773451	1245.290094709	1253.636115837	40.938645411	$\underline{38.937798262}$	40.913156271

## Abbreviations

The following abbreviations are used in this manuscript:

IoT	Internet of Things
NLP	Natural language processing
tf-idf	Term frequency-inverse document frequency
Word2vec	Word-to-vector
LogEvent2vec	LogEvent-to-vector
PCA	Principal component analysis
ICA	Independent component analysis
BGL	BlueGene/L
POP	Parallel log parser
PU Learning	Positive-unlabeled Learning
dLCE	Distributional lexical-contrast embedding model
LSTM	Long short-term memory
k-NN	k-nearest neighbor
SVM	Support vector machine
skip	Gram continuous skip-gram model
cbow	Continuous bag-of-words model
LLNL	Lawrence Livermore National Labs
AUC	Area under curve
ROC	Receiver operating characteristics

## References

[1] Li W., Xu H., Li H., Yang Y., Sharma P.K., Wang J. (2019). Complexity and Algorithms for Superposed Data Uploading Problem in Networks with Smart Devices. IEEE Internet Things J..

[2] Li W., Chen Z., Gao X., Liu W., Wang J. (2019). Multi-Model Framework for Indoor Localization under Mobile Edge Computing Environment. IEEE Internet Things J..

[3] He S., Xie K., Chen W., Zhang D., Wen J. (2018). Energy-aware Routing for SWIPT in Multi-hop Energy-constrained Wireless Network. IEEE Access.

[4] He S., Tang Y., Li Z., Li F., Xie K., Kim H.J., Kim G.J. (2019). Interference-Aware Routing for Difficult Wireless Sensor Network Environment with SWIPT. Sensors.

[5] Wang J., Gao Y., Wang K., Sangaiah A.K., Lim S.J. (2019). An affinity propagation-based self-adaptive clustering method for wireless sensor networks. Sensors.

[6] Wang J., Gu X., Liu W., Sangaiah A.K., Kim H.J. (2019). An empower hamilton loop based data collection algorithm with mobile agent for WSNs. Human-Centric Comput. Inf. Sci..

[7] Wang J., Gao Y., Zhou C., Sherratt S., Wang L. (2020). Optimal coverage multi-path scheduling scheme with multiple mobile sinks for WSNs. Comput. Mater. Cont..

[8] Badshah A., Ghani A., Qureshi M.A., Shamshirband S. (2019). Smart Security Framework for Educational Institutions Using Internet of Things (IoT). Comput. Mater. Cont..

[9] Shi C. (2018). A novel ensemble learning algorithm based on DS evidence theory for IoT security. Comput. Mater. Cont..

[10] Kim D.Y., Min S.D., Kim S. (2019). A DPN (Delegated Proof of Node) Mechanism for Secure Data Transmission in IoT Services. CMC Comput. Mater. Cont..

[11] Park J.S., Youn T.Y., Kim H.B., Rhee K.H., Shin S.U. (2018). Smart Contract-Based Review System for an IoT Data Marketplace. Sensors.

[12] He S., Xie K., Zhou X., Semong T., Wang J. (2019). Multi-Source Reliable Multicast Routing with QoS Constraints of NFV in Edge Computing. Electronics.

[13] Cauteruccio F., Fortino G., Guerrieri A., Liotta A., Mocanu D.C., Perra C., Terracina G., Vega M.T. (2019). Short-long term anomaly detection in wireless sensor networks based on machine learning and multi-parameterized edit distance. Inf. Fusion.

[14] Luo M., Wang K., Cai Z., Liu A., Li Y., Cheang C.F. (2019). Using imbalanced triangle synthetic data for machine learning anomaly detection. Comput. Mater. Cont..

[15] Zhang J., Wang W., Lu C., Wang J., Sangaiah A.K. (2020). Lightweight deep network for traffic sign classification. Ann. Telecommun..

[16] Zhang J., Xie Z., Sun J., Wang J. (2020). A cascaded R-CNN with multiscale attention and imbalanced samples for traffic sign detection. IEEE Access.

[17] Chen Y., Wang J., Xia R., Zhang Q., Cao Z., Yang K. (2019). The visual object tracking algorithm research based on adaptive combination kernel. J. Ambient Intell. Humanized Comput..

[18] Zhou S., Ke M., Luo P. (2019). Multi-camera transfer GAN for person re-identification. J. Vis. Commun. Image Represent..

[19] Xie K., Li X., Wang X., Xie G., Wen J., Cao J., Zhang D. (2017). Fast tensor factorization for accurate internet anomaly detection. IEEE/ACM Trans. Netw. (TON).

[20] Xie K., Li X., Wang X., Cao J., Xie G., Wen J., Zhang D., Qin Z. (2018). On-line anomaly detection with high accuracy. IEEE/ACM Trans. Netw..

[21] Zhu H., Meng F., Rho S., Li M., Wang J., Liu S., Jiang F. (2019). Long Short Term Memory Networks Based Anomaly Detection for KPIs. Comput. Mater. Cont..

[22] Wang Y., Cao Y., Zhang L., Zhang H., Ohriniuc R., Wang G., Cheng R. (2019). YATA: Yet Another Proposal for Traffic Analysis and Anomaly Detection. Comput. Mater. Cont..

[23] Oliva A.F., Perez F.M., Berna-Martinez J.V., Ortega M.A. (2019). Non-deterministic outlier detection method based on the variable precision rough set model. Comput. Syst. Sci. Eng..

[24] Zhu C., Zhao W., Li Q., Li P., Da Q. (2019). Network Embedding-Based Anomalous Density Searching for Multi-Group Collaborative Fraudsters Detection in Social Media. Comput. Mater. Cont..

[25] Zhang S., Meng W., Bu J., Yang S., Liu Y., Pei D., Xu J., Chen Y., Dong H., Qu X. Syslog processing for switch failure diagnosis and prediction in datacenter networks. Proceedings of the 2017 IEEE/ACM 25th International Symposium on Quality of Service (IWQoS).

[26] He P., Zhu J., He S., Li J., Lyu M.R. (2017). Towards automated log parsing for large-scale log data analysis. IEEE Trans. Depend. Secure Comput..

[27] He P., Zhu J., Zheng Z., Lyu M.R. Drain: An Online Log Parsing Approach with Fixed Depth Tree. Proceedings of the IEEE International Conference on Web Services.

[28] Teimoortashloo M., Sedigh A.K. (2018). A dynamic independent component analysis approach to fault detection with new statistics. Comput. Syst. Sci. Eng..

[29] Zhang S., Liu Y., Meng W., Luo Z., Bu J., Yang S., Liang P., Pei D., Xu J., Zhang Y. Prefix: Switch failure prediction in datacenter networks. Proceedings of the ACM on Measurement and Analysis of Computing Systems.

[30] Khatuya S., Ganguly N., Basak J., Bharde M., Mitra B. ADELE: Anomaly Detection from Event Log Empiricism. Proceedings of the IEEE INFOCOM 2018-IEEE Conference on Computer Communications.

[31] He S., Zhu J., He P., Lyu M.R. Experience report: system log analysis for anomaly detection. Proceedings of the 2016 IEEE 27th International Symposium on Software Reliability Engineering (ISSRE).

[32] Meng W., Liu Y., Zhang S., Pei D., Dong H., Song L., Luo X. Device-agnostic log anomaly classification with partial labels. Proceedings of the 2018 IEEE/ACM 26th International Symposium on Quality of Service (IWQoS).

[33] Lin Q., Zhang H., Lou J.G., Zhang Y., Chen X. Log clustering based problem identification for online service systems. Proceedings of the 38th International Conference on Software Engineering Companion.

[34] Bertero C., Roy M., Sauvanaud C., Trédan G. Experience report: Log mining using natural language processing and application to anomaly detection. Proceedings of the 2017 IEEE 28th International Symposium on Software Reliability Engineering (ISSRE).

[35] Meng W., Liu Y., Zhu Y., Zhang S., Pei D., Liu Y., Chen Y., Zhang R., Tao S., Sun P. Loganomaly: Unsupervised detection of sequential and quantitative anomalies in unstructured logs. Proceedings of the Twenty-Eighth International Joint Conference on Artificial Intelligence, IJCAI-19.

[36] Mikolov T., Chen K., Corrado G., Dean J. (2013). Efficient estimation of word representations in vector space. arXiv.

[37] Zhu J., He S., Liu J., He P., Xie Q., Zheng Z., Lyu M.R. Tools and benchmarks for automated log parsing. Proceedings of the 2019 IEEE/ACM 41st International Conference on Software Engineering: Software Engineering in Practice (ICSE-SEIP).

[38] Liu Z., Qin T., Guan X., Jiang H., Wang C. (2018). An integrated method for anomaly detection from massive system logs. IEEE Access.

[39] Wu H.C., Luk R.W.P., Wong K.F., Kwok K.L. (2008). Interpreting TF-IDF term weights as making relevance decisions. Acm Trans. Inf. Syst..

[40] Soucy P., Mineau G.W. Beyond TFIDF Weighting for Text Categorization in the Vector Space Model. Proceedings of the the Nineteenth International Joint Conference on Artificial Intelligence.

[41] Nguyen K.A., Schulte im Walde S., Vu N.T. (2016). Integrating Distributional Lexical Contrast into Word Embeddings for Antonym-Synonym Distinction. Proceedings of the 54th Annual Meeting of the Association for Computational Linguistics (Volume 2: Short Papers).

[42] Debnath B., Solaimani M., Gulzar M.A.G., Arora N., Lumezanu C., Xu J., Zong B., Zhang H., Jiang G., Khan L. LogLens: A Real-time Log Analysis System. Proceedings of the 2018 IEEE 38th International Conference on Distributed Computing Systems (ICDCS).

[43] He S., Li Z., Tang Y., Liao Z., Feng L., Lim S.J. (2020). Parameters Compressing in Deep Learning. Comput. Mater. Cont..

[44] Du M., Li F., Zheng G., Srikumar V. Deeplog: Anomaly detection and diagnosis from system logs through deep learning. Proceedings of the 2017 ACM SIGSAC Conference on Computer and Communications Security.

[45] Vinayakumar R., Soman K., Poornachandran P. Long short-term memory based operation log anomaly detection. Proceedings of the 2017 International Conference on Advances in Computing, Communications and Informatics (ICACCI).

[46] Tuor A.R., Baerwolf R., Knowles N., Hutchinson B., Nichols N., Jasper R. Recurrent neural network language models for open vocabulary event-level cyber anomaly detection. Proceedings of the Workshops at the Thirty-Second AAAI Conference on Artificial Intelligence.

[47] Hernandez-Suarez A., Sanchez-Perez G. (2019). Using Twitter Data to Monitor Natural Disaster Social Dynamics: A Recurrent Neural Network Approach with Word Embeddings and Kernel Density Estimation. Sensors.

[48] Lu H., Shi K., Zhu Y. (2018). Sensing Urban Transportation Events from Multi-Channel Social Signals with the Word2vec Fusion Model. Sensors.

[49] Zhou W., Wang H., Sun H., Sun T. (2019). A Method of Short Text Representation Based on the Feature Probability Embedded Vector. Sensors.

[50] Oliner A., Stearley J. What supercomputers say: A study of five system logs. Proceedings of the 37th Annual IEEE/IFIP International Conference on Dependable Systems and Networks (DSN’07).

